# Rather Crude

**Published:** 1888-02

**Authors:** 


### RATHER CRUDE.

The Western Dental Journal, for November, contains an article on "The Green Stain Upon the Teeth," which is a little remarkable from a number of points of view. It opens thus :

"The green fungus stain, from an etiological standpoint, presents certain peculiarities.

"FIRST—There are fungus stains which are not colored green.

"SECOND—There are bacili bacteria *ed id genus* OMNI ! (*sic*) to be obtained ad libitum from the mouth."

Are these the "certain peculiarities" that belong to the green fungus ? The statement might be paraphrased thus :

Man presents certain peculiarities.

FIRST—There are animals which are not men.

SECOND—There are a great many animals in Brazil.

One who is writing upon strictly scientific subjects should be careful of his logic as well as his facts.

The author speaks of a green cell that is sometimes globular and at other times more nearly oval, the green color of which "appears to spread into the surrounding plants," and from these peculiarities, which are common to a great number of cocci, he seems to identify it with *Micrococcus Chlorinus.* It requires a much more elaborate study than this to distinguish almost anything.

The author also speaks of a "vibrio *lactic*" which produces *acetic* acid. We would not willingly discourage research in any one, but until an observer has made some progress in bacteriology he should be a little modest in presenting his inchoate deductions to professional men, and a little editorial supervision should be exercised lest other beginners in scientific study be misled. The same number of *The Western Dental Journal* says editorially: "The school-boy essayists are catching fits, and right they should;" which, if we ignore its grammatical construction, is certainly a wise remark, and one which justifies us in this criticism.

